# Immunocytochemical profiles of inferior colliculus neurons in the rat and their changes with aging

**DOI:** 10.3389/fncir.2012.00068

**Published:** 2012-09-21

**Authors:** Ladislav Ouda, Josef Syka

**Affiliations:** Institute of Experimental Medicine, Academy of Sciences of the Czech RepublicPrague, Czech Republic

**Keywords:** inferior colliculus, GABA, parvalbumin, calbindin, calretinin, aging, rat

## Abstract

The inferior colliculus (IC) plays a strategic role in the central auditory system in relaying and processing acoustical information, and therefore its age-related changes may significantly influence the quality of the auditory function. A very complex processing of acoustical stimuli occurs in the IC, as supported also by the fact that the rat IC contains more neurons than all other subcortical auditory structures combined. GABAergic neurons, which predominantly co-express parvalbumin (PV), are present in the central nucleus of the IC in large numbers and to a lesser extent in the dorsal and external/lateral cortices of the IC. On the other hand, calbindin (CB) and calretinin (CR) are prevalent in the dorsal and external cortices of the IC, with only a few positive neurons in the central nucleus. The relationship between CB and CR expression in the IC and any neurotransmitter system has not yet been well established, but the distribution and morphology of the immunoreactive neurons suggest that they are at least partially non-GABAergic cells. The expression of glutamate decarboxylase (GAD) (a key enzyme for GABA synthesis) and calcium binding proteins (CBPs) in the IC of rats undergoes pronounced changes with aging that involve mostly a decline in protein expression and a decline in the number of immunoreactive neurons. Similar age-related changes in GAD, CB, and CR expression are present in the IC of two rat strains with differently preserved inner ear function up to late senescence (Long-Evans and Fischer 344), which suggests that these changes do not depend exclusively on peripheral deafferentation but are, at least partially, of central origin. These changes may be associated with the age-related deterioration in the processing of the temporal parameters of acoustical stimuli, which is not correlated with hearing threshold shifts, and therefore may contribute to central presbycusis.

## Introduction

The inferior colliculus (IC) occupies a strategic position in the central auditory system. Almost all of the ascending projections from the lower auditory regions (cochlear nuclei, superior olivary complex, and lemniscus lateralis) converge in the central nucleus of the IC, thus making this structure a converging hub of the ascending as well as the descending auditory pathways of the auditory system (Beyerl, 1978; Druga and Syka, 1984a,b; Pollak and Casseday, 1986; Oliver and Huerta, 1992; Druga et al., 1997; Casseday et al., 2002). Simultaneously, the IC operates as a relay structure to the medial geniculate body (MGB) and subsequently to the auditory cortex.

**Figure 1:**
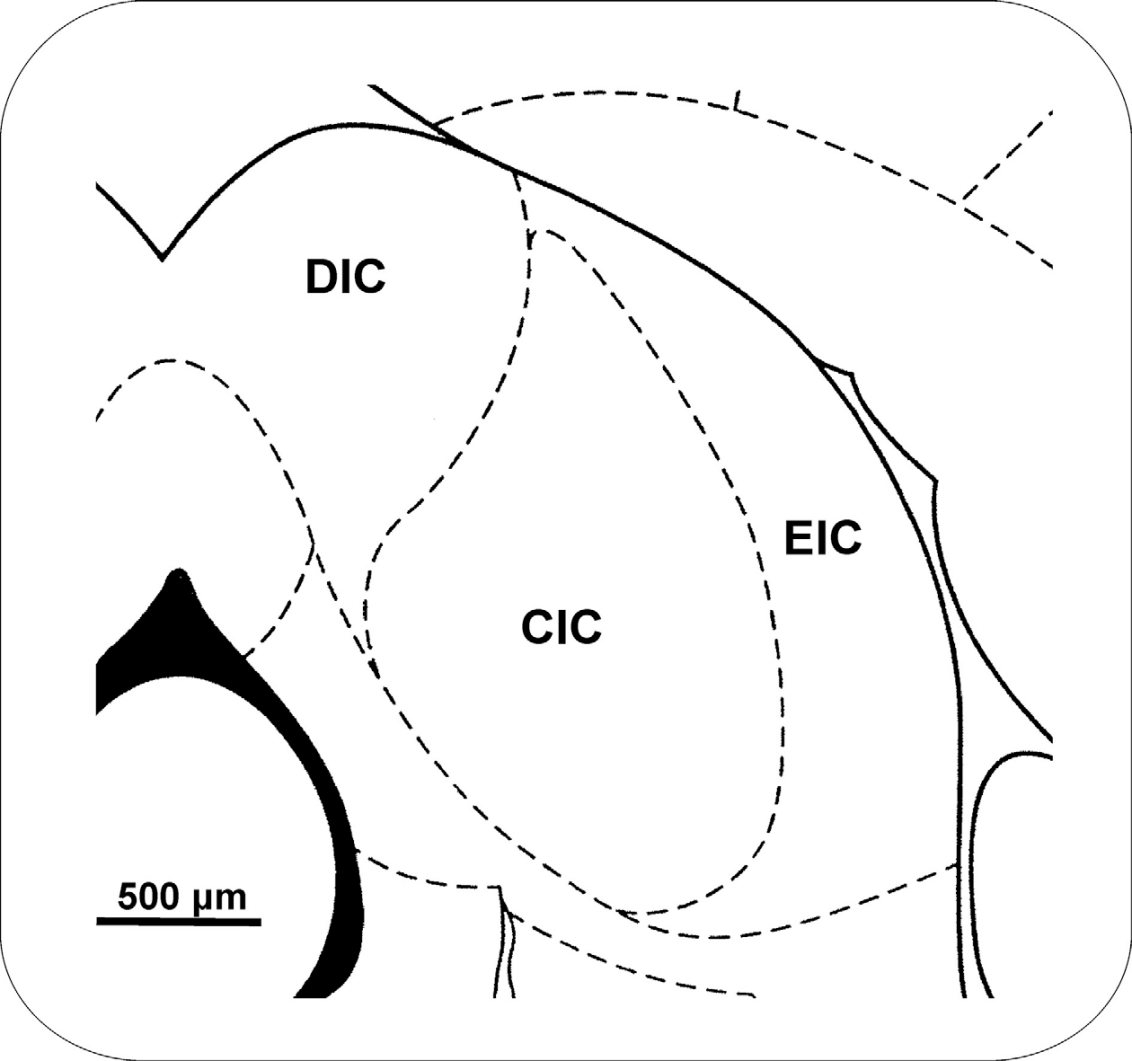
**Topographical schema of a frontal section through the IC.** The drawing is based on Paxinos and Watson (1998). Bregma −8.6 mm. CIC, central nucleus of the inferior colliculus; DIC, dorsal cortex of the IC; EIC, external cortex of the IC.

With respect to its internal composition, the rat IC is subdivided into three major parts, the central nucleus and the dorsal and external (lateral) cortices. This division is visible in most histological and immunohistochemical stainings (Morest and Oliver, 1984; Faye-Lund and Osen, 1985; Malmierca et al., 1993), (Figure 1). In addition, the separation of the ventrolateral nucleus from the external cortex of the IC was proposed, based on homology with the ventrolateral nucleus in the cat (Loftus et al., 2008). The central nucleus of the IC (CIC) represents a part of the primary auditory pathway with a preserved tonotopy, sending primary ascending projections to the ventral subdivision of the MGB and receiving only weak descending projections from the telencephalon (Diamond et al., 1969; Druga and Syka, 1984a,b; Kudo and Nakamura, 1988; Schneiderman et al., 1988). In contrast, the dorsal (DIC) and external (EIC) cortices are more influenced by ascending monoaural pathways and by descending projections from layer V and, to lesser extent, from layer VI of the auditory and non-auditory cortical fields (Druga and Syka, 1984a,b; Saldana et al., 1996; Druga et al., 1997; Winer et al., 1998; Schofield, 2009). The external cortex of the IC possesses strong connections with non-auditory structures including the colliculus superior, substantia nigra, periaquaductal gray, and somatosensory cortex (Syka and Straschill, 1970; Syka and Radil-Weiss, 1971; Aitkin et al., 1978; Druga and Syka, 1984c; Tokunaga et al., 1984; Zhou and Shore, 2006).

Almost the entire GABAergic dorsal nucleus of the lateral lemniscus (Zhang et al., 1998) and the superior paraolivary nucleus (Kulesza and Berrebi, 2000; Saldaña et al., 2009) as well as a substantial portion of neurons from the ventral nucleus of the lateral lemniscus (Riquelme et al., 2001) send ascending inhibitory projections to the neurons of the IC (ipsi- and contralaterally), while excitatory ascending projections originate predominantly from the lateral and medial superior olive and cochlear nuclei (Semple and Aitkin, 1980; Frisina et al., 1998; Malmierca et al., 1998; Riquelme et al., 2001; Davis, 2002; Malmierca et al., 2005). In addition, the contralateral IC is also a source of mostly inhibitory projections to the IC (González-Hernández et al., 1996). GABAergic perisomatic terminals are more prevalent on non-GABAergic neurons in the IC (Merchán et al., 2005).

The strong representation of GABA terminals as well as the high proportion of GABAergic cells in this structure in the rat suggests the presence of strong inhibitory processes in the IC (Gerken, 1996; Batra and Fitzpatrick, 2002; Merchán et al., 2005; Pollak et al., 2011). The GABAergic inhibitory system in the IC serves to modulate the spectral and temporal properties of IC responses with the aim of sharpening the responses to rapid complex sounds (Le Beau et al., 1996; Palombi and Caspary, 1996; Frisina, 2001; Walton et al., 2002).

Besides GABAergic inhibition, also present are significantly weaker glycinergic inhibitory projections onto the IC neurons from various sources (Saint Marie et al., 1989; Saint Marie and Baker, 1990). The presence of glycinergic neurons in the IC is controversial, since Merchán et al. (2005) reported no glycinergic neurons throughout the IC, while Fredrich et al. (2009) observed glycine immunoreactivity in about 1/6 of CIC neurons, however, always in colocalization with GABA. GABAergic puncta were observed both in the neurophil and in the contacts with neuronal somas, whereas very few glycinergic terminals contacted the somata (Merchán et al., 2005).

Auditory function in mammals, including humans, is known to be significantly affected by aging, ultimately resulting in presbycusis with alterations occurring both in the inner ear and in the central auditory system (Syka, 2002; Frisina and Rajan, 2005; Gates and Mills, 2005; Ohlemiller and Frisina, 2008; Frisina, 2009, 2010; Gordon-Salant et al., 2010). The central component of presbycusis is thought to be significantly associated with age-related alterations in the processing of the temporal parameters of complex acoustical stimuli within the central auditory system, especially the IC and auditory cortex (Strouse et al., 1998; Walton et al., 1998, 2002; Frisina and Walton, 2006; Walton et al., 2008; Walton, 2010; Suta et al., 2011). In the human population, a loss of speech understanding with aging constitutes an important health and social impairment (Frisina and Frisina, 1997; Mazelová et al., 2003; Gates and Mills, 2005; Gordon-Salant et al., 2007).

The significance of inhibition for the processing of information occurring in the IC is difficult to overemphasize, as reviewed in detail, e.g., by Pollak et al. (2011). Altered inhibition within the IC of animals may have a severe impact on survival in the wild, since it impairs their ability to refine the localization of a sound source in the environment (Litovsky and Delgutte, 2002; Pecka et al., 2007). In addition, under experimental conditions, the selectivity for features of incoming signals is significantly reduced or eliminated in the majority of IC cells when inhibition is blocked (Casseday et al., 2000; Malmierca et al., 2003; Nataraj and Wenstrup, 2005; Sanchez et al., 2007). Since GABA-mediated inhibition in the IC and auditory cortex is strongly involved in the temporal processing of complex acoustical stimuli, including human language (Walton et al., 1997, 1998; Strouse et al., 1998; Krishna and Semple, 2000; Walton et al., 2002; Suta et al., 2003; Simon et al., 2004), a functional decline in GABA-mediated inhibition may significantly contribute to a deterioration of hearing with aging, i.e., the central component of presbycusis.

In the last few years, we have evaluated age-related changes comprising mostly a decline in the expression of glutamate decarboxylase (GAD) and calcium binding proteins (CBPs) in the higher levels of the rat auditory pathway, including the IC and auditory cortex (Ouda et al., 2008; Burianova et al., 2009; Ouda et al., 2012b). An age-related decline in GABA levels in the IC was previously reported by other authors (Caspary et al., 1990; Gutiérrez et al., 1994; Raza et al., 1994; Caspary et al., 1995). A decreased number of GABA or GAD immunoreactive neurons was also documented in other parts of the rat brain, such as in the hippocampus (Shi et al., 2004; Stanley and Shetty, 2004; Ling et al., 2005), while no significant reduction was found in the sensorimotor or parietal cortex (Poe et al., 2001; Ling et al., 2005; Shi et al., 2006). Age-related changes in the levels of the CBPs in the auditory pathway were also evaluated in the cochlear nuclei and IC of mice. In addition, the reported changes were strain-dependant for all three proteins in the cochlear nuclei (Idrizbegovic et al., 2001, 2004) and for calretinin (CR) in the IC (Zettel et al., 1997).

In the following text, we first discuss in several chapters the quantitative data, the neuronal types and the distribution of particular immunoreactive neuronal populations in the IC of young adult animals. In the second half, we summarize the observed immunohistochemical age-related changes in the IC and their link to electrophysiological and behavioral findings.

## Histochemical profiles of the inferior colliculus in young rats

### Total number of neurons in the IC

The number of neurons in the IC, based on Nissl stained sections, in comparison with other central auditory structures in the rat has been reported in two recent papers (Kulesza et al., 2002; Ouda et al., 2012a). The rat IC contains about 350,000 neurons, with more than 200,000 neurons in the central nucleus alone, about 80,000 in the EIC and 45,000 in the DIC. This represents several times more neurons than in all lower auditory regions combined (cochlear nuclei—30,000 superior olivary complex—16,000–18,000 and lemniscus lateralis—15,000–18,000). In contrast to the IC, the next structure in the auditory pathway, the MGB, contains only about 60,000–70,000 neurons in the rat (with 40,000–45,000 in the ventral division of the MGB), which represents a large difference in comparison to the IC.

In this respect, the position of the MGB may be specific to some degree in the rodent, while the number of neurons in the MGB was reported to be significantly species-dependent, with markedly higher numbers in carnivores and especially in humans (Dorph-Petersen et al., 2009; Najdzion et al., 2011). On the other hand, the absolute number of neurons in the IC of rats and humans is suprisingly comparable, 350,000 vs cca 400,000 neurons (Sharma et al., 2009—a 5-month-old baby that died of postoperative complications after cardiac surgery). In addition, the total number of neurons in the rat auditory cortex is probably roughly the same as in the rat IC (Te1, Te2, Te3—about 600,000 neurons; Ouda et al., 2012a), while, e.g., in primates the divergence of the ascending auditory pathway rapidly increases further through the MGB to the auditory cortical areas. For example, the total number of neurons in the whole human neocortex is almost three orders of magnitude larger than in the whole rat neocortex (Pakkenberg and Gundersen, 1997; Herculano-Houzel and Lent, 2005). These findings strongly support the prominent role of the IC in the rodent central auditory system and suggest that the role of the MGB in rodents may be somewhat different than in other mammalian species.

### Glutamate decarboxylase

**Figure 2:**
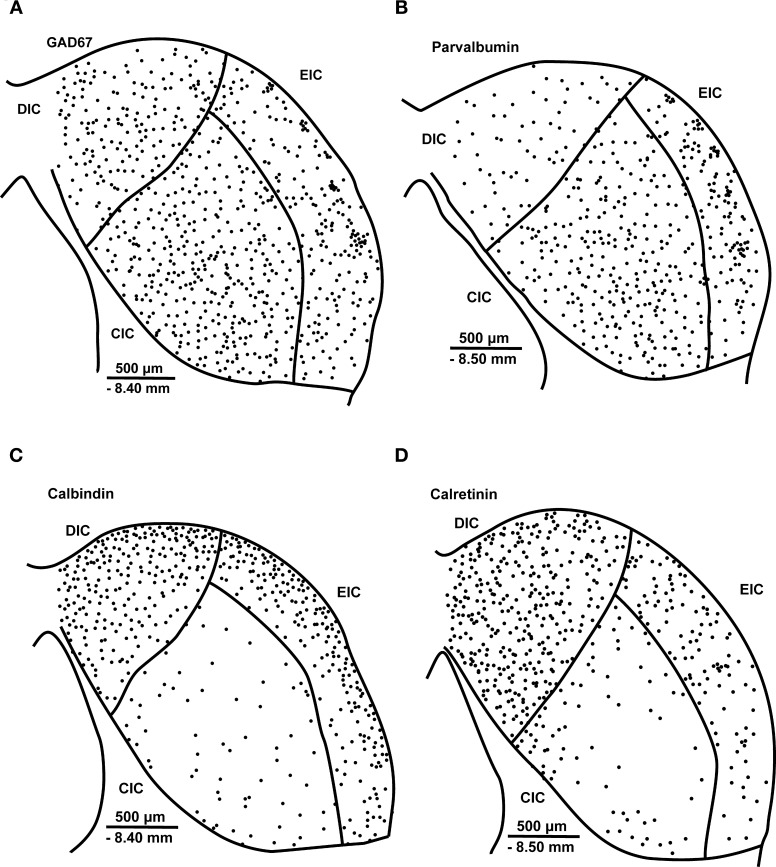
**Schematic illustration of the distribution of glutamate decarboxylase.** GAD67 **(A)**, parvalbumin **(B)**, calbindin **(C)** and calretinin **(D)** immunoreactive neurons in a section containing all three major subdivisions of the IC, the central nucleus and the dorsal and external cortices, in young adult Long-Evans rats. CIC, central nucleus of the inferior colliculus; DIC, dorsal cortex of the inferior colliculus; EIC, external cortex of the inferior colliculus; GAD67, glutamate decarboxylase, isoform 67; PV, parvalbumin; CB, calbindin; CR, calretinin. The distance from bregma and scale bars are provided in each panel. The displayed schema is based on the distribution of GAD67-, PV-, CB-, and CR-ir neurons observed in our previous publications (Ouda et al., 2008; Burianova et al., 2009; Ouda et al., 2012b).

**Figure 3:**
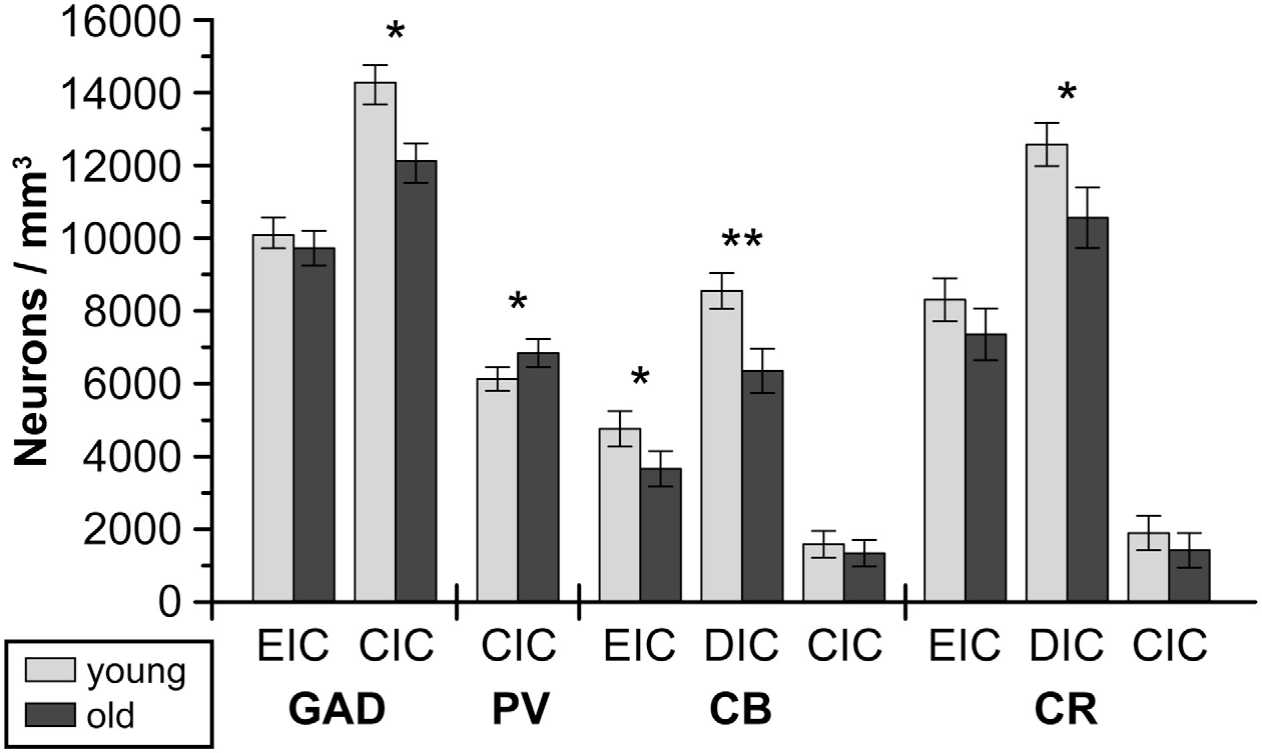
**The numerical density of GAD67-ir, PV-ir, CB-ir and CR-ir neurons in the external and dorsal cortices and in the central nucleus of the IC in young and old Long-Evans rats.** CIC, central nucleus of the inferior colliculus; DIC, dorsal cortex of the inferior colliculus; EIC, external cortex of the inferior colliculus; GAD67, glutamate decarboxylase, isoform 67; PV, parvalbumin; CB, calbindin; CR, calretinin. The displayed results represent a summary of graphs from Ouda et al. (2008); Burianova et al. (2009); Ouda et al. (2012b). The error bars represent S.E.M. (^*^P < 0.05, ^**^P < 0.01).

GABA is synthesized by the decarboxylation of glutamate, and the reaction is catalyzed by the key rate-limiting enzyme GAD. In the mammalian brain, two GAD isoforms of 65 and 67 kDa molecular weight (GAD 65 and GAD 67) are present (Erlander et al., 1991). Most GABA-expressing neurons contain both isoforms, and therefore GAD immunostaining is often used to identify GABAergic neurons (Erlander and Tobin, 1991; Feldblum et al., 1993; Esclapez et al., 1994; Hendrickson et al., 1994). In the rat, GAD65- and 67-immunoreactive(-ir) neurons are distributed throughout all three subdivisions of the IC (Merchán et al., 2005; Burianova et al., 2009) (Figures 2A, 3).

In the CIC of rats, GAD-ir cell bodies of variable sizes are present, relatively homogenously distributed, with a variation in the intensity of immunostaining (Merchán et al., 2005; Burianova et al., 2009). With respect to morphology, the GAD-ir neurons are thought to correspond mostly to the less-flat neurons in the CIC (Malmierca et al., 1993, 1995a), which are homologous to stellate neurons in the CIC of the cat (Oliver, 1984; Oliver et al., 1994). Merchán et al. (2005) proposed that the majority of less-flat neurons are GABAergic, while the majority of flat neurons are excitatory cells (disc-shaped cells in the cat). GABAergic neurons were also reported to be larger on average in their somas when compared to non-GABA neurons in the IC, especially in the CIC (Merchán et al., 2005; Fredrich et al., 2009). In our experiments (Burianova et al., 2009), we did not quantify the size of GAD-ir neurons in comparison to immunonegative ones; however, in terms of morphology, the GAD-ir cells observed in the CIC belonged mostly to the less-flat neurons, with polygonal or oval shapes. In addition, we did not observe any distribution that resembled the location and orientation of the fibrodendritic laminae, which were described to comprise flat cells in the rat (Malmierca et al., 1993) or disc-shaped cells in cat (Morest and Oliver, 1984; Oliver and Morest, 1984).

In the external and dorsal cortices, the identification of morphological types is more complicated. The majority of GAD-ir neurons in the external cortex of the rat IC (EIC) are either large fusiform cells or smaller oval neurons, in both the second and third layers of the EIC. The GAD-ir neurons in the EIC resemble some types described with Rio-Hortega Golgi staining in the rat IC by Malmierca et al. (2011). Especially the large fusiform-like neurons, stained in our sections for GAD (Burianova et al., 2009), might correspond to the second major neuronal type in the EIC, called bitufted neurons with spindle, fusiform somas. In addition, Malmierca et al. (2011) also observed in the second layer of the rat EIC cluster-like groups of small neurons that may correspond to the modules described in detail by Chernock et al. (2004) and found in our own experiments as clusters of GAD-ir (and PV-ir) neurons (Ouda et al., 2008; Burianova et al., 2009). In the rat DIC, the situation is even more complex due to the large heterogeneity of neuronal shapes (Malmierca et al., 2011), which makes direct identification from immunostained sections practically impossible. In our GAD-staining, lower numbers, in comparison to the EIC and CIC, of predominantly small-sized immunoreactive neurons with a homogenous distribution were present in the rat DIC (Burianova et al., 2009).

The clusters of small neurons and intensely stained neuropil in the second layer of the rat EIC described by Chernock et al. (2004) are clearly visible in most sections to the naked eye (Chernock et al., 2004; Burianova et al., 2009). In addition to GAD, the clusters are immunopositive for NADPH-diaphorase and parvalbumin (PV), whereas they are immunonegative for glycine, CB, choline acetyltransferase, and SMI-32. These clusters or modules are absent in the mouse, squirrel, cat, bat, and macaque monkey, and so far they have been found only in rats and were suggested to possibly participate in the animal's spatial orientation (Chernock et al., 2004).

As already mentioned, the GABAergic neurons represent a substantial part of all neurons in the IC. In rats, the portion of inhibitory neurons in the IC seems to be larger than in the cat or bat; specifically; the CIC of the rat contains more than 30% of GABAergic neurons, whereas in the same structure of the cat or the bat they represent approximately 20% (Oliver et al., 1994; Winer et al., 1995; Merchán et al., 2005; Fredrich et al., 2009). The rat DIC and EIC are reported to contain about 20–25% of GABAergic neurons, while the data from other mammalian species are not known (Merchán et al., 2005). In addition to this, the GABAergic projection from the IC to the MGB has been reported to be more prominent in the rat than in some non-rodent species (Peruzzi et al., 1997; Ito et al., 2009). The reason for the difference from the known results in other mammalian orders is not clear. However, the next structure in the rat ascending auditory pathway, the MGB, contains almost no inhibitory neurons in the rat in contrast to other mammals. In the rat MGB, GABAergic neurons represent less than 1% of cells, while they form a significant portion (25%) of all neurons in the cat or primate MGB (Winer and Larue, 1996). Therefore, the only inhibition in the rat MGB is of extrinsic origin, which might be linked to the higher representation of GABAergic inhibition and projection in the rat IC.

The brainstem auditory nuclei, especially the dorsal and ventral nuclei of the lateral lemniscus as well as the superior paraolivary nucleus, have generally been considered to be a major source of inhibitory inputs to the neurons of the IC (ipsi- and contralaterally) (Zhang et al., 1998; Kulesza and Berrebi, 2000; Riquelme et al., 2001; Saldaña et al., 2009). However, the number of rat CIC GABAergic neurons, i.e., 30% of 200,000–230,000, which are either local circuit neurons or projecting neurons sending local collaterals within the IC (Oliver et al., 1991; Malmierca et al., 1995b; Ito et al., 2009), is markedly higher than the number of GABAergic neurons in all the lower brainstem auditory nuclei in total. Therefore, the most important source of GABAergic terminals on the rat IC neurons might be the intrinsic GABAergic IC neurons themselves.

Recently, two types of GABAergic neurons were described in the IC of the rat with different synaptic organization and axonal projections: smaller GABAergic neurons, which are predominatly local circuit interneurons, and larger GABAergic neurons, predominantly projecting to the MGB (Ito et al., 2009). The projecting GABAergic neurons are more prevalent in the central nucleus of the IC and are densely covered perisomatically with glutamatergic synaptic contacts that could allow them to fire easily and rapidly. The exclusive presence of vesicular glutamate transporter 2 in presynaptic terminals suggests that the predominant sources of these synaptic connections are either collateral branches of IC glutamatergic neurons or fusiform cells in the dorsal cochlear nucleus. In addition, they possess very thick axons which may allow them to deliver inhibitory input to the MGB in advance of excitatory inputs from the glutamatergic IC neurons firing simultaneously (Ito et al., 2009).

In our previous study, we concluded that a minor neuronal population of about 10% of the neurons in all three major subdivisions of the IC of the rat expresses non-phosphorylated neurofilaments labeled by SMI-32 antibody (Ouda et al., 2012a). The SMI-32 antibody was shown to specifically label a subset of cortical and subcortical neurons in the mammalian brain, predominantly those with long and thick axonal projections (Sternberger and Sternberger, 1983; Kirkcaldie et al., 2002; Voelker et al., 2004; Molnár and Cheung, 2006). The non-phosporylated neurofilament content in neurons is thought to be associated with the large extent of axonal myelination and with large neuronal size (Campbell and Morrison, 1989; Tsang et al., 2000; Kirkcaldie et al., 2002). Since the GABAergic neurons in the rat IC are reported to be larger than non-GABAergic cells (Merchán et al., 2005), and simultaneously projection GABAergic neurons in the CIC and EIC, containing thick myelinated axons, are thought to be larger than non-projecting GABAergic neurons (Ito et al., 2009), we might speculate that the non-phosphorylated neurofilaments labeled by SMI-32 in the IC are preferentially expressed in a subset of the larger projecting GABA-ergic neuronal subpopulation. However, a double labeling study is necessary to confirm such a hypothesis.

### Calcium binding proteins

The calcium binding proteins (CBPs), CB, PV, and CR, represent major fast cytoplasmatic calcium buffers in the central nervous system and thus protect neurons from insults that induce an elevation of intracellular Ca^2+^ (Baimbridge et al., 1992; Elston and González-Albo, 2003). The disruption of neuronal calcium homeostasis and consequent molecular events affect neuronal viability and synaptic plasticity and may represent early steps in the development of neuronal degeneration (Foster, 2007). Changes in calcium homeostasis in the brain during aging or during different pathologies are thought to be tightly linked to a decline in neuronal performance (Khachaturian, 1989; Verkhratsky and Toescu, 1998; Toescu et al., 2004).

The distribution of calbindin-immunoreactive (CB-ir), calretinin immunoreactive (CR-ir) and parvalbumin-immunoreactive (PV-ir) neurons in the central nervous system of the rat was first described in summary by Celio (1990) and Résibois and Rogers (1992). Their distribution seems to be complementary to some extent with the major occurrence of PV in the primary (tonotopic) and CB and CR in the non-primary rat auditory regions (Lohmann and Friauf, 1996), as also described in mice (Cruikshank et al., 2001), chinchillas (Kelley et al., 1992), monkeys (Jones, 2003), and humans (Tardif et al., 2003; Sharma et al., 2009). Specifically in the IC, CB-ir, and CR-ir neurons are abundant in the dorsal and external cortices and relatively rare in the central nucleus of the IC, while PV-ir neurons are present in all three parts of the IC, with a predominant distribution in the CIC. The mutual colocalization of CB, PV, and CR in one neuron is known to be relatively rare, as they mainly represent three distinct neuronal populations; in the higher parts of the auditory pathway their expression is mostly found (in the case of PV almost exclusively) in GABAergic neurons (Demeulemeester et al., 1989; Kubota et al., 1994; Gonchar and Burkhalter, 1997; Jinno and Kosaka, 2002; Markram et al., 2004; Gonchar et al., 2007).

### Parvalbumin

PV-ir neurons are present in all three subdivisions of the rat IC (Lohmann and Friauf, 1996; Ouda et al., 2008). In the CIC, neurons with a large variation in the size of their neuronal somas are rather uniformly distributed (Figure 2B). In our PV-ir staining, the intensity of their immunoreactivity varied from low to high and their morphology resembles the less-flat cells, similarly as in GAD immunostaining. In the EIC, the majority of PV-ir neurons belonged to the large spindle-like multipolar cells and smaller oval neurons (Ouda et al., 2008). The clusters of PV-immunoreactive somas and neuropil in the second layer of the EIC are very similar to the clusters in GAD immunostaining and visible in practically all examined rats, as in GAD immunostaining (Chernock et al., 2004; Ouda et al., 2008). The DIC contains a relatively low number of mostly oval, small-sized PV-immunoreactive neurons (Lohmann and Friauf, 1996; Ouda et al., 2008).

In the neocortex, the PV-expressing neurons mostly form a well-defined population of fast-spiking GABA-ergic basket cells that are essential for the generation of gamma oscillations, which are thought to provide a temporal framework for information processing in the brain (Kawaguchi and Kubota, 1998; Bartos et al., 2007). These neurons comprise roughly 40–50% of all cortical GABAergic neurons; practically all PV-ir cortical cells are also GABAergic cells (Gonchar and Burkhalter, 1997; Gonchar et al., 2007). The co-expression of GAD and the functional importance of the PV-ir neuronal population in the IC are known to a lesser extent. From our experiments (Ouda et al., 2008), we can estimate that the number of GAD-ir neurons in the rat IC is roughly two times higher in the central nucleus and external cortex of the IC than the number of PV-ir neurons (Figure 3). On the other hand, Fredrich et al. (2009) identified almost all GAD-ir neurons in the central nucleus of the rat IC as co-expressing PV. The reason for this discrepancy is not clear; however, the different antibodies used and variations in the immunoreactivity of PV-ir somas pivotal for classifying a cell as PV-positive might be responsible to some degree for this difference. The potential preference for PV expression in some GABAergic subpopulations, especially larger GABAergic neurons projecting to the MGB vs. smaller local circuit interneurons (Ito et al., 2009), is open to further investigation. However, the PV-ir neurons in the CIC varied in size from small to large-sized in our staining, similarly as for GAD immunostaining (Ouda et al., 2008; Burianova et al., 2009), which may suggest the presence of PV in both of the above-mentioned GABAergic subpopulations.

In any case, the distribution of PV-ir neurons in the whole IC reflects relatively well the distribution of GABAergic (GAD-ir) neurons (compare Figures 2A and 2B). Furthermore, the morphological types, at least of the neuronal somas, present in our immunostainings correspond relatively well to the same classification of GAD-ir neurons discussed in the previous section on GAD. In addition, the similar appearance of clusters in the EIC in both GAD and PV immunostaining (Chernock et al., 2004) supports the idea of a strong overlap of GAD and PV immunoreactivity in the IC. In addition, only one group of cellular clusters in the second layer of the EIC was evaluated by Malmierca et al. (2011) with Rio-Hortega Golgi staining. However, the precise extent of these overlaps, even in the discussed clusters in the second layer of the EIC, requires a double labeling study to determine, which has not yet been performed in the EIC.

### Calbindin

CB-ir neurons are present in all three subdivisions of the rat IC; however, the majority of CB-ir cells are found in the dorsal and external cortices, while only a few weakly stained neurons are scattered throughout the central nucleus of the IC (Förster and Illing, 2000; Ouda et al., 2012b) (Figure 2C).

A relatively homogenous neuropil and a high numerical density of CB-ir neurons are typical for the rat DIC, with a gradual decrease from the cortical surface toward the borders with the central nucleus. The neuronal somata are mainly oval or polygonal with loosely stained dendrites (Ouda et al., 2012b). In the rat EIC, the numerical density of immunoreactive cells is lower (Figure 3) and decreases toward the CIC and toward the basal part of the IC. The cellular morphology in the EIC is more heterogenous with oval, elongated or triangular somas. No signs of cluster-like immunoreactivity are present in the EIC (Chernock et al., 2004; Ouda et al., 2012b). In both the DIC and CIC, the highest numerical density of CB-ir neurons can be found adjacent to the surface of the IC and decreases toward the central nucleus. The CIC is demarcated as an area with low CB neuropil staining and weakly stained sparse CB-ir neurons (Förster and Illing, 2000; Ouda et al., 2012b).

As mentioned previously, CBPs were reported to be expressed mostly by GABAergic neurons (Freund and Buzsáki, 1996; Gonchar and Burkhalter, 1997; Gonchar et al., 2007); however, calbindin most likely does not follow this rule. For example, some weakly stained CB-ir cells in the superficial cortical layers of the neocortex are known to be pyramidal neurons (Kubota et al., 1994; De Felipe, 1997). In the IC, the notable prevalence of calbindin (and also calretinin) in the dorsal and external cortices and its low occurrence in the CIC suggest that at least some of these neurons are not GABAergic. This suggestion is further supported by the fact that in our quantitative estimates, we found that the numerical density of calbindin- and calretinin-immunoreactive neurons in the DIC is even higher than the number of all GAD-ir neurons in the DIC (unpublished data). In addition, clusters of smaller oval neurons that are GAD-ir and PV-ir in the EIC are not visible in calbindin immunostaining (Ouda et al., 2012b). No double labeling study in the DIC or EIC has been undertaken so far, while in the CIC, Fredrich et al. (2009) did not report the colocalization of GABA and CB/CR using double staining.

### Calretinin

CR-ir neurons are found in all three subdivisions of the rat IC with a significant presence in the dorsal and external cortices (Lohmann and Friauf, 1996; Ouda et al., 2012b) (Figure 2D). In the CIC, a weaker positivity of the neuropil and a lower numerical density of CR-ir neurons are distinctive features, while in the DIC, a relatively homogenous neuropil and a very high numerical density of CR-ir neurons are dominant features (Ouda et al., 2012b).

In the rat EIC, an interesting feature of CR immunostaining that we could often find in our sections stained for calretinin is a strip-like distribution of CR-ir neurons and neuropil positivity throughout all three cortical layers, with patches with a higher concentration of immunoreactive neuropil and neurons that are surrounded by less stained neuropil. One might speculate about a link to the clusters evaluated by Chernock et al. (2004) that display GAD, parvalbumin and NADPH-diaphorase immunoreactivity. However, these *pro tempore* called strips or patches are not delineated as clearly and consistently or with such contrast as the clusters in GAD or PV immunostaining; furthermore, they are larger and situated deeper from the IC surface across the EIC (Figure 2D). Dual labeling will be necessary to determine any such potential relationship. The form of the CR-ir neuronal somas in the EIC ranges from small-sized to large neurons and oval or polygonal in shape, while a few cells with spindle-like morphology are present (Ouda et al., 2012b).

The correspondence of calretinin-expressing neurons with particular morphological types and specifically with the GABA-mediated system in the IC is more ambiguous than in the case of parvalbumin. As stated above, the numerical density of calbindin- and calretinin-immunoreactive neurons in the DIC is even higher than the number of all GAD-ir neurons in the DIC. However, in contrast to calbindin, the presence of a few large spindle-like neurons and potential cluster-like immunoreactivity suggests a more pronounced overlap with the population of GAD-ir neurons, at least in the external cortex of the IC.

Interestingly, the distribution and numerical density of CR-ir neurons throughout the IC are similar to the distribution and numerical density of neurons positive for NADPHdiaphorase(-d), which identifies neurons capable of producing nitric oxide (Dawson et al., 1991; Hope et al., 1991; Druga and Syka, 1993; Loftus et al., 2008; Wu et al., 2008). In our estimation, the numerical density of NADPH-d-positive neurons in all three major divisions of the IC is close to the numerical density of CR-ir neurons (unpublished data). In the IC, the NADPH-d-positive neuronal subpopulation is known to be predominantly glutamatergic, while only a minority of NAPDH-diaphorase-positive cells in the IC is GABAergic neurons (Wu et al., 2008). No double labeling study of CR and NADPH-d was reported in the IC; however, a substantial colocalization of CR and NADPH-d expression in neurons was observed in the hippocampus and periaqueductal gray (Czéh et al., 2005; Barbaresi et al., 2012). On the other hand, the pattern of clusters in the second layer of the EIC labeled by NADPH-d staining apparently corresponds to the pattern of the clusters present in GAD and PV immunostaining (Chernock et al., 2004).

## Influence of aging on the expression of immunocytochemical markers

The inferior colliculus, similarly as other structures of the auditory pathway in the rat, undergoes essential changes with aging. To demonstrate these changes clearly, we decided to use for aging studies two rat strains (the inbred Fischer 344 strain and the outbred Long-Evans strain) with a wide variety of morphological, physiological and behavioral differences, including differently preserved hearing function with age (for review see Syka, 2010). For example, similarly as with other inbred strains, Fischer 344 rats display large cognitive deficits in different tests of spatial memory in the Morris water maze in contrast to wild rats and Long-Evans rats (Harker and Whishaw, 2002). While Long-Evans rats represent a strain with relatively well preserved peripheral hearing function up to late senescence, a rapid and pronounced deterioration of hearing function with aging is found in Fischer 344 rats, resulting in larger hearing threshold shifts, a decrease in the amplitude of click-evoked auditory brainstem responses, a diminution of distortion product otoacoustic emissions and a decrease in middle-ear compliance (Popelar et al., 2003, 2006). Age-related sensory deficits in F344 rats also include visual function, which is damaged due to retinal degeneration (Di Loreto et al., 1994). In spite of the differences between strains, we found that age-related changes in the immunocytochemical markers in the IC were almost identical in both strains.

### Glutamate decarboxylase

**Figure 4:**
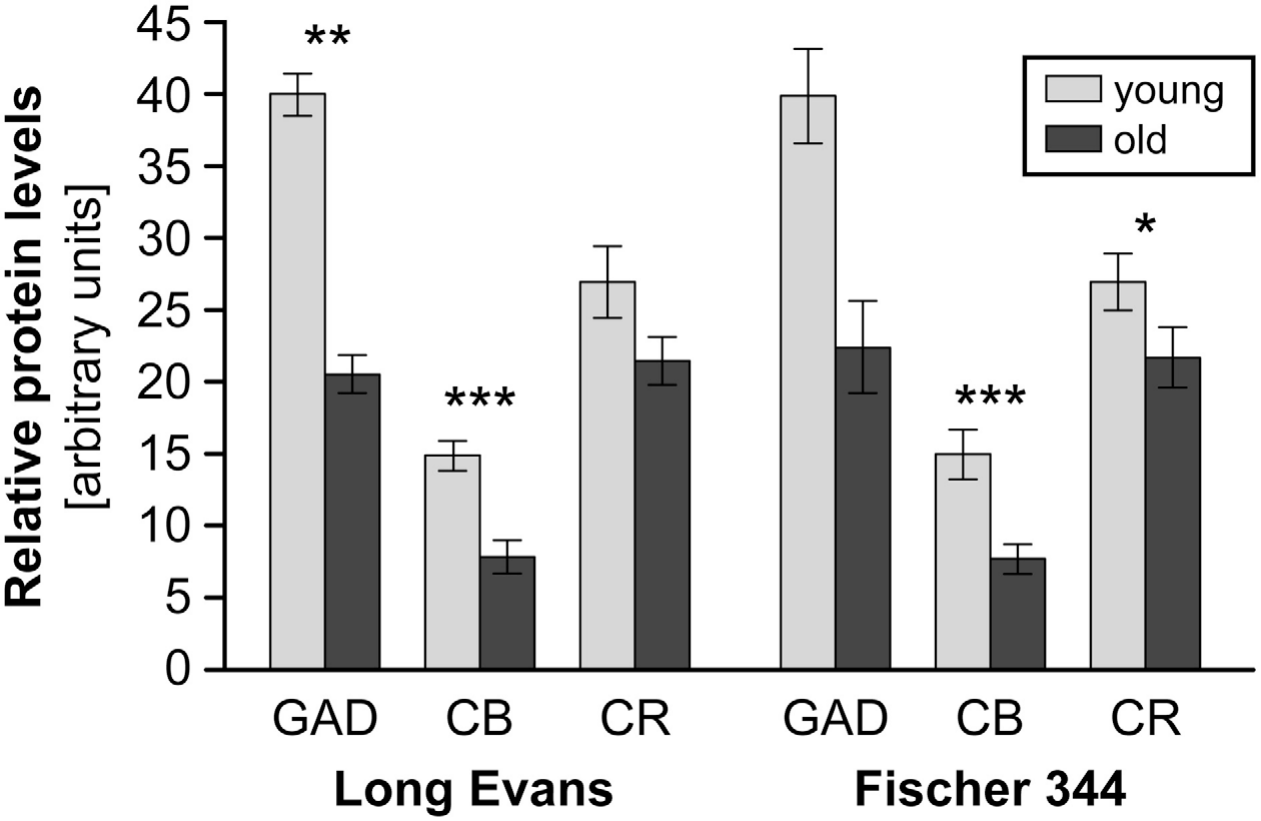
**The results of western blot protein analysis of GAD67, calbindin (CB) and calretinin (CR) in the IC of young and old Long-Evans and Fischer 344 rats.** Arbitrary units were calculated as the ratio between the optical density of the examined protein and the optical density of actin (in scanned films analysed using ImageQuant software). The heights of the columns for GAD67, CB, and CR cannot be mutually compared; they are dependent on the ratio of protein levels between the IC and the cortical areas for each examined protein. The tall column for GAD67 (in arbitrary units) indicates a larger amount of GAD67 per unit tissue weight in the IC when compared to the cortex (and conversely for CB). Due to the limited number of old Fischer 344 rats available for GAD67 protein analysis, the data did not demonstrate statistically significant differences in this case. The graph is constructed from data presented in Burianova et al. (2009); Ouda et al. (2012b). The error bars represent S.E.M. (^*^P < 0.05, ^**^P < 0.01, ^***^P < 0.001).

In GAD-65 and 67 immunoreactivity (Burianova et al., 2009), significant declines with aging were observed in the CIC and EIC comprising a decrease in the optical density of GAD-ir neuronal somas and a decrease in the number of GAD-ir cells (CIC only, Figure 3). These findings were supported by the results of western blot analysis that demonstrated a significant age-related decline of about 50% in the levels of GAD65 and GAD67 proteins in the IC of old rats in comparison with young animals (Figure 4). The analyzed samples for western blotting included the whole IC (i.e., all three subdivisions of the IC at once). The decline in GAD immunoreactivity was also similar in the auditory cortex, and the pattern of changes was very similar in both Long-Evans and Fischer 344 animals. Interestingly, the protein levels of both isoforms of glutamate decarboxylase (GAD65 and 67) were found to be approximately threefold higher (per unit of tissue weight) in the rat IC in comparison with the neocortical areas in both young and old animals (Sheikh et al., 1999; Burianova et al., 2009), which might further support a prominent role for inhibition in the rat IC.

An age-related decrease in the number of GABA-immunoreactive cells associated with a decrease in enzymatic activity, the levels of GABA and the release of GABA in the rat CIC was also reported by other authors (Caspary et al., 1990, 1995; Gutiérrez et al., 1994; Raza et al., 1994). Simultaneously, GABA-A and GABA-B receptor binding intensity was reported to decline, and the protein levels of the receptor subunits were found to be altered during aging in the IC of Sprague-Dawley and Fischer 344 rats (Gutiérrez et al., 1994; Milbrandt et al., 1994; Caspary et al., 1999; Schmidt et al., 2010). The compromised GABA-mediated inhibition in the IC could consequently result in a broadening of the excitatory areas and thus in the poorer tuning of the neuronal receptive fields. In turn, the reduction of fine-tuned receptive fields resulted in a poorer discrimination of the temporal parameters of sounds, as also demonstrated by electrophysiological data in the IC (Palombi and Caspary, 1996; Walton et al., 1998, 2002; Simon et al., 2004).

### Calcium binding proteins

Despite the almost exclusive colocalization of GABA-ir and PV-ir in neurons in the IC and neocortex (Gonchar and Burkhalter, 1997; Gonchar et al., 2007; Fredrich et al., 2009), the age-related changes in parvalbumin immunoreactivity were different when compared with changes in GAD 65 and 67 (Ouda et al., 2008). In old Long Evans rats, the observed changes were rather mild: the number of PV-ir neurons in the CIC was slightly increased, associated with an increase in the optical density of PV-ir somas and a slight decrease in the mean neuronal volumes. In contrast, in old Fischer 344 rats a non-significant tendency toward a decrease in the number of PV-ir neurons in the IC and significantly smaller mean neuronal volumes were present. The observed changes could not be attributed to any specific type of neuron. In contrast to these findings, a significant reduction in the occurrence of PV-ir neurons was found in the auditory cortex of Fischer 344 animals with aging, whereas in Long Evans rats such a reduction was not observed.

The age-related changes in calbindin and calretinin immunoreactivity had a rather similar pattern and were relatively uniform, regardless of the examined rat strain or structure (IC and auditory cortex). We found similar significant age-related changes in CB immunoreactivity in the dorsal and external cortices of both Long Evans and Fischer 344 rats. The changes included a decrease in the number of CB-ir neurons and a significant decline in the average volumes of CB-ir neuronal somas (DIC only). In western blotting, the age-related changes were even more pronounced and resulted in a significant decline in the levels of calbindin in the whole IC of old rats of both species of almost 50%. In calretinin immunoreactivity, a tendency toward an age-related decline in the number of CR-ir neurons as well as a significant decrease in the mean volumes of the remaining CR-ir neuronal somas were found in the dorsal and external cortices of the IC. Western blot analysis demonstrated a significant decline, however, less pronounced when compared to calbindin and GAD, in calretinin protein levels of 22% in both rat strains (Ouda et al., 2012b). Summaries of the immunohistochemical age-related findings and western blot results are presented in Figures 3, 4.

In an inter-species comparison, strain-dependent differences were found in the inferior colliculus of the CBA/CaJ and C57/BL/6J mouse strains: with aging, both strains showed a decline in the number of calbindin-ir neurons in the IC, similarly as in our study. However, the number of CR-ir neurons did not change with age in C57 mice and even significantly increased in the IC of CBA mice (a mouse strain with preserved hearing function up to an advanced age) (Zettel et al., 1997). In the case of calretinin, the changes were found to be activity-dependent, since early bilateral deafening prevented calretinin up-regulation in the DIC of aged CBA animals (Zettel et al., 2001). This difference from the findings in Long-Evans rats might reflect inter-species differences to some degree. A decline in the number of calcium binding protein (CBP)-ir neurons were observed in different brain neocortical areas in humans, including the auditory cortex, without any previous signs of psychiatric or neurological diseases (Bu et al., 2003). Increasing evidence has also accumulated about the mutual links among intracellular calcium regulation, alterations in intracellular CBP levels and neurodegenerative and neuropsychiatric disorders (for review see Woo and Lu, 2006; Mattson, 2007). The disruption of calcium homeostasis in neurons may consequently be involved in both the impairments that accompany normal aging and also in the different pathologies associated with age-related disorders (Khachaturian, 1989; Foster, 2007; Mattson, 2007; Riascos et al., 2011). In this context, it may be surprising that in mutant mice (CB-/-, CR-/- or PV-/-), the deleted individual CBP is not compensated for by the up-regulated expression of any other CBP (Schwaller et al., 2002). Nevertheless, the mice (of all three types) are not only able to survive, but actually display no significant changes in phenotype, either in the general morphology of the nervous system or their behavior under normal housing conditions. The morphological and functional changes are rather subtle and manifest during specific behavioral experiments, which may suggest a more complicated function for CBPs in neurons, not only as simple calcium buffers and thus as neuroprotective molecules, but also as complex components in intracellular calcium homeostasis involved in the subtle regulation and timing of calcium signals pre- or postsynaptically (Schwaller et al., 2002; Schwaller, 2010).

### Changes in the total number of neurons

The decreased number of immunoreactive neurons (GAD-, CB-, CR-ir, etc.,) in the IC observed in aging studies cannot be easily attributable to a general neuronal loss, because in our previous experiments we found that the reduction in the total number of neurons in the IC of old Long-Evans and Fischer 344 rats in Nissl-stained sections does not exceed 10% (unpublished data). This finding is in agreement with the data of other authors, who reported no significant changes accompanying aging in the total number of neurons in the rat inferior colliculus (Helfert et al., 1999) or other rat brain regions, including the hippocampus and cortical areas (Merrill et al., 2001; Poe et al., 2001; Stanley and Shetty, 2004). Specifically, in the IC of old Fischer 344 rats, the number of both inhibitory and excitatory synaptic terminals decreased while no age-related reduction in the total number of neurons was found (Helfert et al., 1999). For comparison, a stereological study demonstrated an age-related decrease in the total number of neurons in the human neocortex of fewer than 10% (Pakkenberg and Gundersen, 1997).

Therefore, the diminished expression of proteins in originally immunoreactive neurons may be the reason for the age-related reduction in the detectable numbers of immunoreactive neurons. Similarly, up-regulated or *de novo* expression in previously non-expressing neurons may be the reason for the increase in the number of immunoreactive neurons in situations such as the reported increase of CR-ir neurons in the IC of old CBA mice (Zettel et al., 1997).

### The link to electrophysiological and behavioral data

So far, it has not been known whether the age-related changes in the IC (and/or auditory cortex) are mainly the consequence of decreased ascending inputs from the deteriorated periphery or rather part of the changes in the aging brain itself. Most immunohistochemical experiments concerning age-related changes in the GABA-mediated inhibition of central auditory system were undertaken in animal strains with a pronounced hearing loss and deterioration of the peripheral sensory organ with aging, which represented a complication for determining the cause of the observed changes (for review see Frisina, 2001, 2009, 2010; Syka, 2002; Caspary et al., 2008). In our experiments, the age-related decline in the number of GAD-ir, CB-ir, and CR-ir neurons as well as the decline in GAD, CB, and CR protein levels in the inferior colliculus (and auditory cortex) were found to be largely independent of the peripheral deterioration, including the levels of the hearing threshold shifts (Burianova et al., 2009; Ouda et al., 2012b).

Hearing function was shown to be altered in the absence of any significant damage to the peripheral sensory organ, since the age-related worsening of gap detection thresholds, the gap duration difference limen and the disappearing middle latency response to an increasing stimulus repetition rate are not correlated with hearing threshold shifts in Long-Evans rats (Suta et al., 2011). Specifically, the gap detection thresholds were shown to be based predominantly on subcortical structures, since the induced temporary inactivation of the left auditory cortex in the rat by the administration of muscimol resulted in a significant worsening of the gap duration difference limen, while gap detection thresholds were not significantly changed (Rybalko et al., 2010). Furthermore, the neural code necessary for behavioral gap detection is present in the temporal discharge patterns of the majority of IC neurons (Walton et al., 1997).

Similarly, in electrophysiological studies on aging Fischer 344 rats, the reduction in the maximum discharge rate or the increased number of units responding poorly to an auditory stimulus in the IC was not correlated with the observed hearing threshold shift. In addition, for spontaneous activity, first spike latency or dynamic range, no significant declines were observed with aging (Palombi and Caspary, 1996). Also in mice, spontaneous rates and the distribution of temporal discharge patterns did not differ significantly between young and old CBA/CaJ mice, and the age-related decline in the processing of temporal sound features or the coding of envelope periodicities was suggested to be likely related to a dysbalance between inhibitory and excitatory neuronal mechanisms (Walton et al., 1998, 2002). Significant central functional age-related hearing alterations in the IC were found in both the CBA/CaJ mouse strain, with relatively preserved peripheral hearing up to late senescence, and the C57/BL/6J mouse strain, which serve as a model of rapid and severe sensorineural presbycusis, though the particular character of the changes was different (Walton et al., 1998, 2002; Felix and Portfors, 2007).

In a recent age-related study, (Rybalko et al., in press) performed in our laboratory, the acoustic startle reflex (ASR) (a transient motor response to an intense unexpected stimulus) and the prepulse inhibition (PPI) were used as an indicator of the behavioral responsiveness to sound stimuli. While the ASR is an unconditioned reflex reaction, in the PPI procedure, the startle reaction is inhibited by a low-level sound that shortly precedes the intense startle stimulus (Davis et al., 1982; Koch, 1999). Animal studies have shown that in the auditory system, PPI is involved in the cochlear nucleus, the inferior and superior colliculi and the pedunculopontine tegmental nucleus (Nodal and López, 2003), with the cortex and hippocampus exhibiting a modulatory effect on the PPI (Koch, 1999). The PPI of ASR, which represents a basic inhibitory process regulating sensory inputs, can be used for estimating age-related changes in inhibitory function (Geyer and Braff, 1987; Swerdlow et al., 2001). In our experiments, both Long-Evans and Fischer 344 rats exhibited a similar age-related progression of PPI deterioration, with a significantly decreased inhibitory efficacy in aged rats. This decrease was not correlated with the observed hearing threshold shifts.

Therefore, we may suggest that the age-related decline in GAD expression in the rat IC and AC, as demonstrated in our experiments, does not depend exclusively on peripheral deafferentation but is, at least partially, of central origin. The age-related alterations in the IC involving the GABA-mediated inhibitory system and the decline in the number of neurons expressing glutamate decarboxylase and CBPs may significantly contribute to the observed behavioral impairment.

### The reversibility of changes and functional implications

The potential long-term reversibility or compensation of the age-related changes in the expression of glutamate decarboxylase or CBPs is open to further investigation. However, it is known that changes in the expression of selected neuronal markers in the IC neurons, including CBPs that are induced by experimental manipulations may be reversible to some extent.

For example, following monoaural cochlear ablation in the ferret, the CR-ir plexus in the contralateral CIC increased its optical density and the area of immunostaining (Fuentes-Santamaria et al., 2003). The increase in CR immunostaining was interpreted as an up-regulation of CR expression in a subset of IC afferents. In rats, a similar increase in the levels of CR was found after unilateral enucleation in the contralateral superior colliculus (Arai et al., 1993). Furthermore, the induced changes were found to be reversible to some degree after a longer time period. A unilateral ablation of the auditory cortex in rats resulted in a progressive increase in the number and optical density of CR-ir neurons in the dorsal and external cortices, i.e., the subdivisions of the IC more inervated by the cortico-collicular projection, which returned to control values during the long-term survival of the animals (Clarkson et al., 2010). Similarly, the number of NADPH-d-positive neurons in the inferior colliculus declined significantly after a lesion of the auditory cortex at short survival intervals in rats; however, this reduction diminished after longer survival intervals (Druga and Syka, 2001). The reversible character of the changes in protein expression supports the idea of the preservation of the affected neurons.

With respect to studies on aging, similar data are very limited. At the cortical level, a partial recovery of age-related deterioration was shown by De Villers-Sidani et al. (2010). The age-related hearing deficits and the decline in the number of PV-ir neurons in the auditory cortex of old rats were partially reversed after intensive auditory behavioral training. Similarly, in the visual cortex of old monkeys, deteriorated GABA-mediated function was observed with aging, while the local administration of GABA temporarily improved the visual function of the neurons (Leventhal et al., 2003). Nevertheless, despite peripheral deterioration that leads to reduced input thoughout the auditory pathway to the IC and central neurochemical changes affecting the levels of neurotransmitters or CBPs, the compensatory mechanisms are probably highly active in sensory systems in aged animals and humans, as also emphasized by other authors (Palombi and Caspary, 1996; Syka, 2002; Caspary et al., 2008; Frisina, 2010).

## Conclusions

The inferior colliculus may play an even more prominent role in the central auditory system of rats than usually thought, since the rat IC contains more neurons in total than all other subcortical auditory structures combined (Kulesza et al., 2002; Ouda et al., 2012a). In addition, inhibitory neurons in the rat IC, including GABAergic projections from the IC to the MGB, are present in abundant numbers when compared to the cat or bat, whereas intrinsic GABAergic neurons are practically absent in the MGB of rats and other rodents in contrast to other mammalian orders (Winer and Larue, 1996; Merchán et al., 2005; Ito et al., 2009).

Along with the auditory cortex, the IC plays a key role in the processing of the temporal parameters of sounds, which is strongly dependent on the function of the inhibitory systems. Both temporal parameter processing and GABA-mediated inhibition are known to be altered in aged rats (Syka, 2002; Caspary et al., 2008; Frisina, 2010). The decline in the number of neurons expressing glutamate decarboxylase and CBPs as well as the decline in the levels of these proteins occuring in the IC with aging, as observed in our experiments, could represent a basis for central functional deterioration (Ouda et al., 2008, 2012b; Burianova et al., 2009). Since the total number of neurons in the IC likely does not decline significantly with aging (Helfert et al., 1999), the diminished expression of proteins in originally immunoreactive neurons may be the reason for the age-related reduction in the detectable numbers of immunoreactive neurons. Furthemore, the age-related decline in the number of immunoreactive neurons and the levels of the proteins occurring in the central auditory system is not exclusively dependent on the loss of peripheral inputs, but may represent, at least partially, a feature of the aging brain and significantly contribute to the deterioration of hearing function known as central presbycusis.

Central presbycusis represents a complex process that includes alterations present in the subcortical auditory nuclei, the auditory cortex and other non-auditory regions, as well as the development of partial compensatory mechanisms. The potential reversibility or compensation of these age-related changes is open to further investigation. A better understanding of the mechanisms underlying impaired neuronal processing in the central auditory system with aging, resulting in the poor recognition of complex environmental sounds, species-specific vocalizations in animals and speech in man, may have important implications for the treatment or amelioration of the negative aspects of presbycusis in the future.

### Conflict of interest statement

The authors declare that the research was conducted in the absence of any commercial or financial relationships that could be construed as a potential conflict of interest.
